# Removal of Diethylhexyl Phthalate from Hands by Handwashing: Evidence from Experimental N-of-1 and Crossover Designs

**DOI:** 10.1038/s41598-017-00581-2

**Published:** 2017-03-28

**Authors:** Pi-I. D. Lin, Chia-Fang Wu, Hwang-Shang Kou, Tzu-Ying Huang, Jentaie Shiea, Ming-Tsang Wu

**Affiliations:** 1 Kaohsiung Medical University, Department of Public Health, Kaohsiung, 807 Taiwan; 2 Kaohsiung Medical University, Research Center of Environmental Health, Kaohsiung, 807 Taiwan; 3 Harvard T.H. Chan School of Public Health, Department of Environmental Health, Boston, 02115 USA; 4 Kaohsiung Medical University, Department of Pharmacy, Kaohsiung, 807 Taiwan; 5 National Sun Yat-Sen University, Department of Chemistry, Kaohsiung, 807 Taiwan; 6 Kaohsiung Medical University Hospital, Department of Family Medicine, Kaohsiung, 807 Taiwan; 7 Kaohsiung Municipal Hsiao-Kang Hospital, Center of Environmental and Occupational Medicine, Kaohsiung, 807 Taiwan

## Abstract

Phthalate exposure through skin is often neglected due to the small quantity and limited dermal absorption rate. However, free phthalate can be ingested by hand-to-mouth action or by contact with food. To evaluate the effectiveness in removing phthalate exposure on hand, we compare here the removal efficiency of di-(2-ethylhexyl)phthalate (DEHP) on hands by handwashing with soap-and-water versus water-only. In two three-day N-of-1 trials, residual DEHP was measured in a single female adult who washed exposed hands with soap-and-water or water-only. Subsequently, a crossover study was performed by randomly assigning another 28 subjects equally to wash with soap-and-water or with water-only, and then each one received the other treatment 24 hrs later. In the N-of-1 trials, mean DEHP removal rates range from 95.9% (SD = 0.1%) to 97.0% (SD = 2.5%) for soap-and-water handwashes, and 1.8% (SD = 0.1%) to 7.0% (SD = 0.3%) (n = 3) for water-only. In the crossover study, mean removal rate was 94.6% (SD = 6.5%) for handwashing with soap-and-water (n = 28) and 8.7% (SD = 5.7%) for water-only (n = 28). We concluded that handwashing with soap-and-water removes 80% more DEHP than handwashing with water alone, and may be a cost-effective way of removing other endocrine disruptors from hands.

## Introduction

Handwashing is an important and cost-effective public health intervention for the prevention of infectious diseases spread^1–3^. Despite its known benefits for reducing the global burden of diseases causing diarrhea, only 19% of the world population washes hands with soap after contact with excreta^4^. Even though handwashing has been demonstrated to remove chemicals on hands in occupational settings^5, 6^ and frequent hand-washers were found to have lower chemical exposure levels according to an observational study^7^, public health campaign seldom shed lights on the benefit of handwashing beyond the scope of infectious diseases. In this study, we aimed to provide evidence for the effectiveness of handwashing in reducing exposure to phthalate.

Phthalates, a family of chemicals used in plastics and other products, is one of the most prevalent toxic chemical and endocrine disrupting chemicals to which humans are exposed in their daily lives. Phthalates are known to disrupt endocrine function and adversely affect sex and thyroid hormones, reproductive function, and neurodevelopment, particularly in infants, toddlers, and children^8–11^. Exposure to these chemicals is difficult to avoid due to their ubiquitous prevalence in the environment, where exposure occurs through the ingestion of contaminated foods, direct skin contact^12, 13^, and transdermal absorption from air^14^. Being lipophilic, phthalates are easily picked up by hands through contact with products such as PVC flooring, personal care products, and plastic toys and subsequently enter to bodies via dermal absorption or hand-to-mouth routes such as finger sucking, nail biting or smoking^15, 16^. Toddlers and children are more susceptible to phthalate exposure due to their floor play and hand-to-mouth behaviors. In fact, children’s exposure to diethylhexyl phthalate (DEHP), a potent, commonly used phthalate, from toys and hand-to-mouth activities was estimated to be 5.7–44 μg/kg bw/day using child-specific exposure factors from the US Environmental Protection Agency (EPA)^17, 18^. This level had exceeded the adult reference dose (RfD) of 20 μg/Kg bw/day set by US EPA^19^.

Dermal exposure accounts for approximately 10–30% of DEHP exposure in children^13, 15, 18^. Effective interventions to reduce dermal exposure can help lower the overall phthalate exposure level among children. A study in Taiwan found that handwashing is one of the most important strategies in reducing phthalate metabolites in urine among girls^20^. In this study, we demonstrated the effectiveness of handwashing in removing phthalate from hands by employing thorough experimental designs with N-of-1 trials and a crossover study, comparing soap-and-water versus water-only handwashing to reduce phthalate contamination on hands.

## Results

### N-of-1 Trials

The 25-year-old female subject had a body mass index (BMI) of 20.4, body weight of 46.0 kg, hand breadth of 9.2 cm, and hand length of 16.1 cm. She completed two 3-day experimental N-of-1 trials. The water and room temperatures (mean ± SD) were 24.4 ± 0.2 °C and 24.9 ± 0.5 °C, respectively (n = 3) (Table 1).

**Table 1:** Summary of the subject and environmental characteristics in two interventional studies.

Study Design	N-of-1	Crossover
N or Mean ± SD		
N	1	28
Female	1	14
Male	—	14
Age (yrs)	25.0	22.7 ± 2.9
Female	25.0	23.5 ± 3.7
Male	—	21.9 ± 1.6
BMI	20.4	22.3 ± 3.5
Female	20.4	20.2 ± 2.4
male	—	24.4 ± 3.1
Body weight (kg)	46.0	62.6 ± 14.3
Female	46.0.4	52.0 ± 6.8
Male	—	73.3 ± 11.7
Hand length (cm)	18.1	18.1 ± 1.2
Female	18.1	17.5 ± 1.0
Male	—	18.8 ± 0.9
Hand breadth (cm)	7.2	8.1 ± 0.7
Female	7.2	7.6 ± 0.4
Male	—	8.6 ± 0.4
Water temperature (°C)	24.4 ± 0.2	25.8 ± 1.3
Water-only	24.5 ± 0.2	25.9 ± 1.2
Soap-and-water	24.4 ± 0.2	25.8 ± 1.3

**Figure 1:**
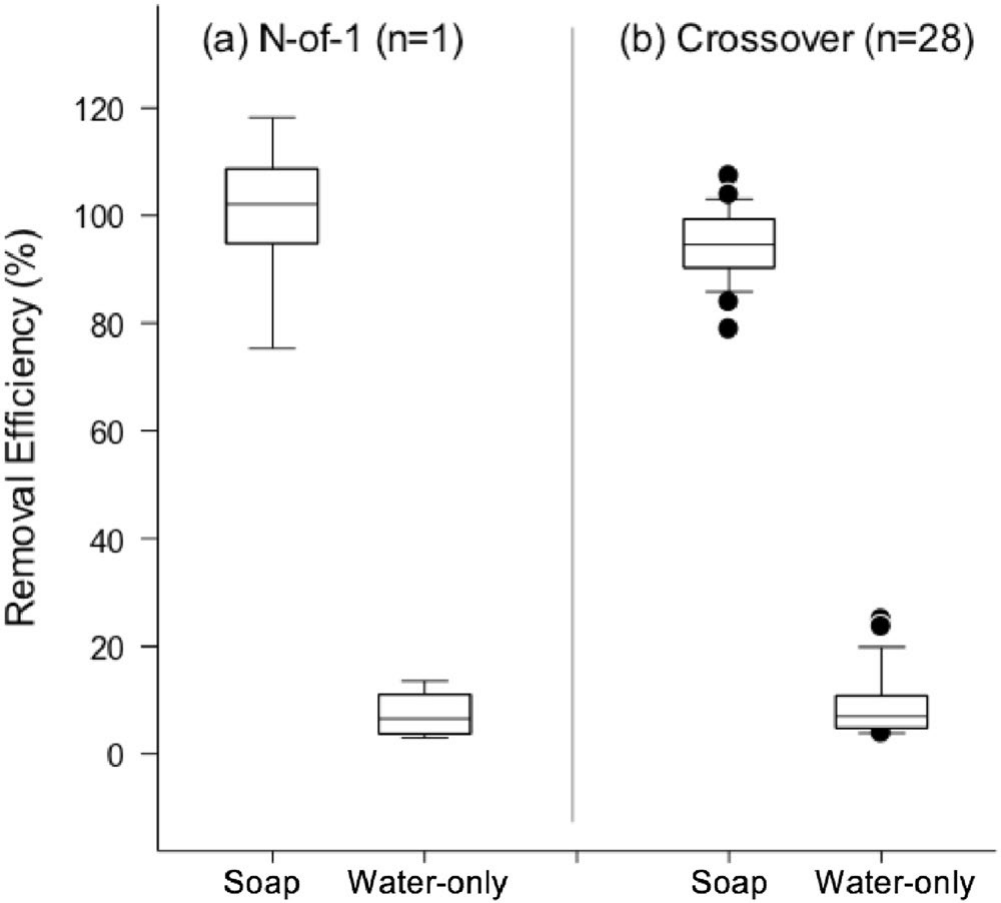
Box-Whisker plot showing the removal rate of dermal DEHP exposure of both hands in each study. (**a**) Average of the two N-of-1 trials on one female subject; (**b**) The crossover study on 28 study subjects.

**Figure 2:**
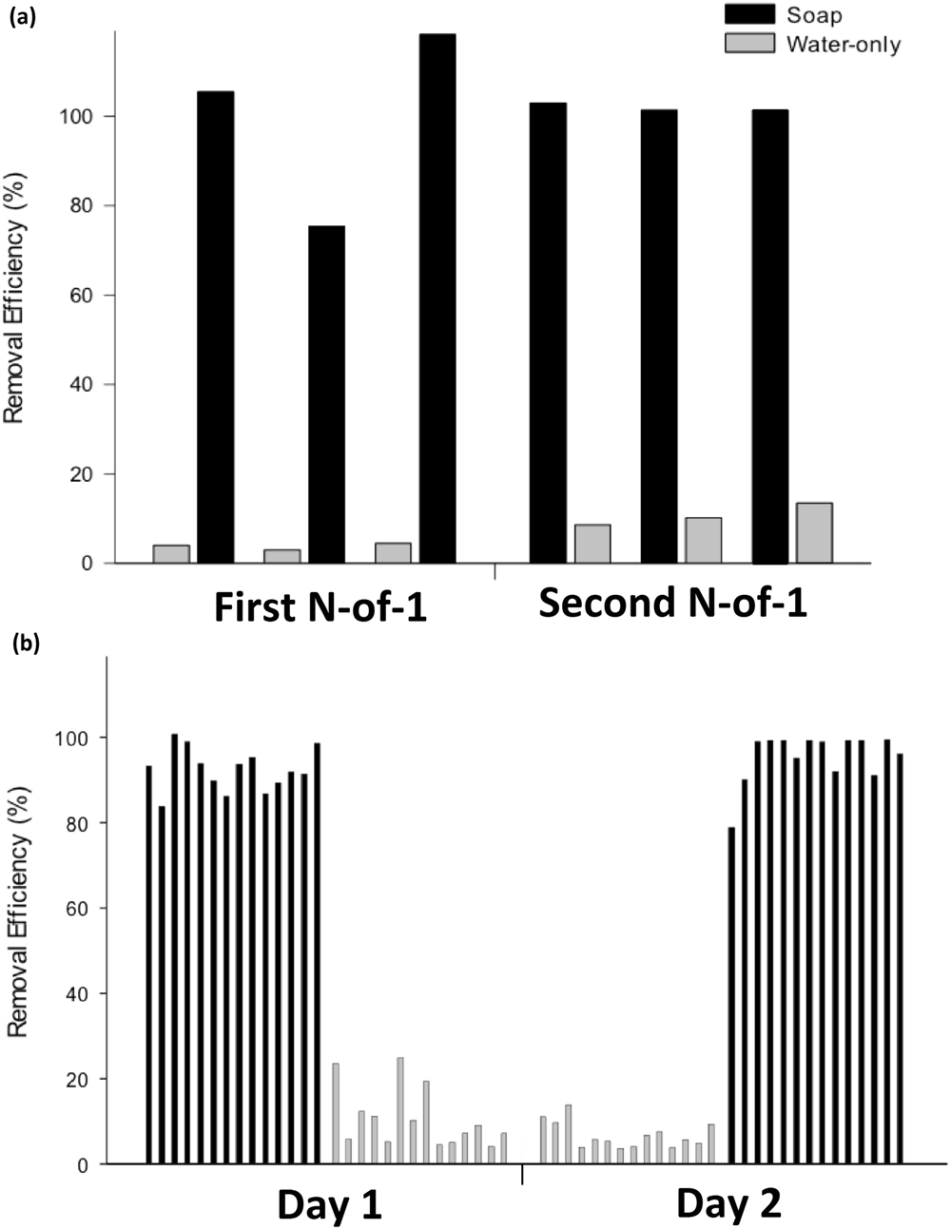
Removal rate of dermal DEHP exposure over both hands in each individual. (**a**) N-of-1 trials on one female subject; (**b**) The crossover study on 28 study subjects. The removal rate accomplished by handwashing with soap and water (black) or water only (gray) for each individual whose hands were exposed to 0.5 mL of 1000 μg/mL standard DEHP solution.

In the first three-day trial, the DEHP removal rate (mean ± SD) was 1.8 ± 0.4% by the water-only handwash and 97.0 ± 13.3% by the soap-and-water handwash (n = 3). In the second 3-day trial, the DEHP removal rate by soap-and-water handwash (mean ± SD) was 95.9 ± 0.1%, and the water-only handwash removal rate was 7.0 ± 0.3% (n = 3). The average removal of DEPH by the soap-and-water wash was significantly higher than the water-only wash (P < 0.001) (Fig. 1a). Compared to that in the first 3-day trial, the variability of the DEHP removal rate by soap-and-water treatment in the second 3-day trial among the three days was smaller (SD = 6.5 for first 3-day and SD = 6.4 for second 3-day), suggesting a possible learning effect in the course of experimental trials (Fig. 2).

### Crossover Study

The participants consisted of 28 healthy people (14 females, 14 males; aged 22.7 ± 2.9 yrs). The mean (±SD) hand length and breadth (cm) were 18.1 ± 1.2 and 8.1 ± 0.7, respectively; male subjects had significantly larger hands compared to female subjects (P < 0.05). The mean water and room temperatures were consistently similar throughout our study (Table 1).

The mean DEHP removal rates were 94.6 ± 6.5% for handwashing with soap-and-water and 8.7 ± 5.7% for handwashing with water-only. The removal efficiency was significantly different between the two types of handwashing methods (P < 0.001) (Fig. 1b). Among the water-only handwashes, a subsequent handwashing with soap-and-water successfully removed most of the DEHP residuals on hand (additional 70.6 ± 11.6% of DEHP removed). No statistically significant difference in removal efficiency was found between male and female subjects for both the soap-and-water and the water-only handwashes (data not shown). There was no significant inter-day trial difference, though a significant borderline improvement was found in day two of the water-only group (P = 0.06) (Table 2). This was likely due to the repeated learning bias, and the learning effect was more evident among males.

**Table 2:** Comparison of the removal rate of DEHP by handwashing between the intervention and placebo group in the crossover study (N = 28).

	Removal rate (%)^1^			
	Average	Day 1	Day 2	P-value
Soap and water (Intervention)	94.64 ± 6.49	96.04 ± 6.50	93.25 ± 6.40	0.263
Water only (Placebo)	8.74 ± 5.69	6.72 ± 3.12	10.76 ± 6.98	0.059
p-value	<0.001	<0.001	<0.001	

[^1^Mean ± standard deviation].

## Discussion

DEHP was removed much more efficiently by handwashing with soap-and-water than with water alone. On average, one handwashing with soap-and-water removed more than 90% of DEHP in our study, whereas one handwashing with water alone removed only 10%. The poor removal efficiency achieved by handwashing one time with only water was substantially improved by combined soap-and-water handwashing. Compared to a previous study^15^ which did not employ a standardized Six-Step Hand Hygiene Technique (60–75% DEHP removed by handwashing), the present study found a higher DEHP removal efficacy by using a standardized Six-Step Hand Hygiene Technique. This previous study with only 5 participants had similar handwashing procedure (eg. the application of soap, the washing duration, and hand drying time) as our study, except the handwashing method, where participants were only asked to rub hands together using typical “washing” pattern. The Six-Step Hand Hygiene Technique ensured that all surface areas of the hands were well washed. The result of the study provided concrete evidences on the benefits and efficacy of handwashing with soap using such standardized technique.

Because children are much more vulnerable to the effects of phthalates and are harder to control, we chose college students as subjects in our interventional studies. We compensated for the difference in subjects by designing an experiment to mimic the behavior of dermal exposure to DEHP in toddlers and children. To do this, we chose 500 μg as the exposure dose of DEHP in our interventional studies. The average surface area of one hand in children aged 6–11 years was estimated to be 510 cm^2^ ^21^. Based on the mean dermal DEHP exposure (0.571 μg/cm^2^) measured by Kim and his colleagues^13^, we estimated that the total amount of DEHP on both hands would be approximately 582.42 μg (510 cm^2^/hand × 0.571 μg/cm^2^ × 2 hands) in children, close to the exposure dose of 500 μg in the present study. Another reason for choosing this exposure level was for comparative purposes. Standardized guidelines have been established to assess dermal exposure in occupational and residential settings, such as the EPA OPPTS 875.2400 test guideline^22^. In a study with comparable exposure conditions, where four workers were exposed to 500 μg of Mancozeb, a hydrophobic pesticide, by 0.5 mL spiking on their hands, the wash efficiency with soap-and-water was 77 ± 3%^5^. Thus, our interventional studies reflected the actual DEHP exposure dose among children in real-life situations. However, considering adult’s larger hand surface area compared to children, the surface loading (mass of chemicals per skin surface area) for children may be higher. This could result in overestimation of the removal efficiency when using adult participants to estimate children’s removal efficiency. We calculated the level of overestimation using the removal efficiency of DEHP in hand area with different DEHP exposure levels provided by Gong *et al*.^15^, and found that when the exposure level was 43% higher (in microgram/meter square), the removal efficiency reduced about 23% (see detailed calculation in the Supplementary appendix).

Handwashing involves three main forces to remove contaminants from skin surfaces: mechanical action, hydrodynamic drag, and wet chemical action^5^. Rinsing hands involves mostly hydrodynamic drag. Handwashing with water-only, which involved scrubbing the skin via mechanical agitation through back-to-forward movements and pressure of both hands, consisted of mechanical force and hydrodynamic drag. Handwashing with soap-and-water, however, not only involved mechanical action and hydrodynamic drag but also wet chemical (soap) action. The non-polar ends of the dispersed soap bond with hydrophobic chemicals, such as DEHP, and form micelles that can be easily rinsed off the skin. In the N-of-1 trials and the crossover study, one time handwashing with water-alone removed only 5% of the DEHP, whereas handwashing with soap-and-water removed more than 70% DEHP. These results suggest that wet chemical action (dissolution) is the most important force contributing to the removal of lipophilic chemicals such as phthalates^5^.

The removal rate of DEHP in some of N-of-1 trials by the treatment of soap-and-water may exceed 100%. Possible explanations are that DEHP exposure traces may be left on the subject’s hand before the start of the experiment which was not removed by the prewash step, or simply due to experimental variation. Although we asked the subject to thoroughly wash her hands before the start of the experiment, in this first N-of-1 trial, she might not be as familiar with the Six-Step Hand Hygiene Technique and thus may not have washed all surface area on hand and removed all of the possible DEHP exposure prior to the start of the trial. This also highlights the learning effect of the handwashing technique on improving removal efficiency, which was observed in both the N-of-1 and the crossover experiments.

As DEHP does not hydrolyze in water, the types of tensides within the soap are important in DEHP removal. In our pilot study, we tested major brands of antimicrobial soap, plain handmade soap (vegetable oil and sodium hydroxide), and unscented soaps and found that they worked equally well (unpublished data). Even though we only used one type of phthalate in the study, considering that many phthalate esters have similar chemical and physical characteristics, our results can be generalized to phthalate esters or other chemicals with similar bonding affinity to human skin.

In conclusion, a one-time wash with soap-and-water using a standardized Six-Step Hand Hygiene Technique removes most of the DEHP on hands and is better than washing with water-only. Because DEHP is not readily absorbed through the skin, it is likely that exposure would occur hand-to-mouth. Therefore, we recommend that people, especially toddlers and children, wash their hands properly with soap and water at least shortly before eating.

## Methods

### Study Designs

**Figure 3:**
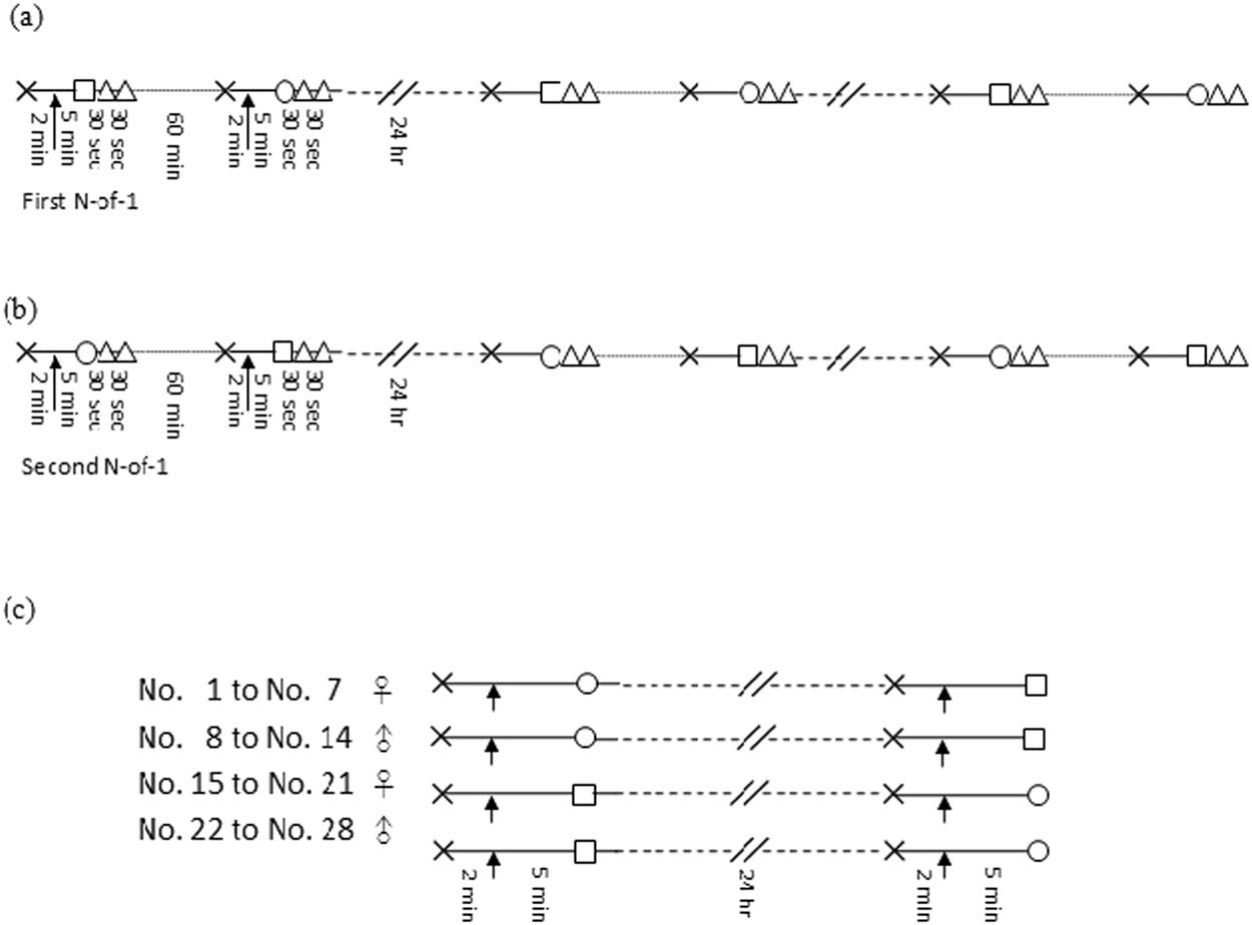
Flowchart of the different study designs. (**a**) First experimental episode of the N-of-1 on one female subject; (**b**) Second experimental episode of the N-of-1 on one female subject (**c**) Crossover study on 28 subjects. The interventional studies included prewash with water rinsing (cross), then direct dispersing of 0.5 mL of 1000 μg/mL standard DEHP solution evenly over both hands (arrow), handwashing with soap and water (circle) or water only (square), and additional water rinsing (triangle). A washout period of 60 min was implemented between each handwashing intervention, and repeats of the intervention pairs were separated by 24 hrs.

The study was registered at ClinicalTrials.gov (http://www.clinicaltrials.gov, identifier: NCT02707172). Three trials, consisting of two N-of-1 trials and one crossover study, were used to compare the effect of handwashing with soap-and-water and handwashing with water-only on phthalate removal from hands (Fig. 3). The soap was plain soap, consisting of vegetable oil and sodium hydroxide. First, we recruited one healthy female aged 25 yrs to participate in two N-of-1 trials^23, 24^. In each trial, a trained research technician placed 500 μg of DEHP on the participant’s hands and asked the participants to thoroughly spread DEHP over both hands. The participant was then asked to wash hands with soap-and-water or water-only. The trials were repeated for three times over the course of three days. The first trial started on 15 May 2013. On Day One, after the subject prewashed her hands, she allowed her hands to be exposed to a designated amount of DEHP. She then washed them with water-only and rinsed them sequentially in three separate polyethylene (PE) bags, each containing 200 mL water to collect DEHP residues. One hour later, the same protocol was followed, except the exposed hands were treated with soap-and-water instead of water-only (Fig. 3a). The water rinsing in three separate bags remained the same. The pairs of studies were repeated on two subsequent days (16 and 17 May). In the second N-of-1 trial, which was also performed over three days (22–24 May), the protocol using the same subject remained the same for all three days, except that the subject underwent soap-and-water handwash first then water-only handwash (Fig. 3b). The water rinsing remained the same.

A randomized 2 × 2 crossover study^25^ was subsequently conducted between June and September 2013. We assigned one group of 7 females and 7 males to perform handwashing with soap-and-water and another group of 7 females and 7 males to perform handwashing with water-only (Fig. 3c). Briefly, on the first day, the two groups washed their DEHP exposed hands using either soap-and-water or water-only and then rinsed them in a PE bag with 200 mL water to collect DEHP. After 24 hrs, the experiment was crossed-over with the same two groups receiving the opposite treatments.

All protocols were approved by the the IRB at Kaohsiung Medical University Hospital (Approval No. KMUHIRB-2013-02-03(II)). The methods were carried out in accordance with the approved guidelines. Informed consent for the experiments was obtained from all subjects. Participants’ names and other HIPAA identifiers were all removed during the study.

### Standardized DEHP Exposure and Handwashing Protocol

Before each N-of-1 trial or crossover study was run, subjects washed both hands thoroughly with soap and a large amount of tap water in front of a sink. After they had completely dried their hands under cool air using an air blower for approximately 2 min, they rinsed them in 200 mL double deionized water in a PE bag (Ziploc®, SC Johnson & Son, Wisconsin, US) for 30 sec. They allowed water to drip off their hands inside the bag above the water for another 10 sec and then flicked all digits inside the bag above the water five times to remove excess water. The water collected in this bag was later measured for DEHP. For both of the N-of-1 trials and the crossover study, the DEHP concentrations in the water collected from the prewash were non-detectable.

After the prewash step, subjects completely dried both hands under cool air from an air blower for approximately 2 min. Then, 0.5 mL of 1000 μg/mL DEHP solution (total of 500 μg DEHP, dissolved in 50% methanol) was dispersed evenly over both hands using the same pipette. The subjects rubbed their hands together to spread the solution thoroughly over the surface of both hands, including both thumbs, all eight fingers, and the palmar and dorsal areas of the palms above the wrist. They let their hands dry in the air briefly. Subsequently, 1000 μL of soap-and-water (500 μL soap solution +500 μL water) or water-only (1000 μL water), both kept in separate 45-mL amber-colored glass bottles for blinding, was applied by a research technician to the hands of the subject, who washed them following the Six-Step Hand Hygiene Technique^26, 27^. The clear color liquid soap (KLEENEX® Fragrance & Dye-Free Skin Cleanse, Kimberly-Clark, Georgia, USA) and water (Milli-Q® purified water, Millipore, Massachusetts, US) were indistinguishable in appearance before lathering (eFig. 1).

### Six-Step Hand Hygiene Procedure

The Six-Step Hand Hygiene Technique was performed for a total of 30 sec, with 5 sec per step^26, 27^. After washing their hands, the subjects thoroughly rinsed both hands above the wrist in 200 mL double deionized water in a PE bag for 30 sec. They allowed the water of both hands held inside the bag above the water to drip into the bag for another 10 sec, and then they flicked all digits inside the bag above the water five times to remove excess water.

All of the samples in the collected bags were transferred to 20-mL phthalate-free amber-colored glass bottles (eFig. 1) and stored in a refrigerator at 4 °C until analysis by high-performance liquid chromatography (HPLC) equipped with an ultraviolet (UV) detector, which were described in a Supplementary Appendix in detail (eFig. 2)^28, 29^. All stored samples were analyzed within 3 days.

### Statistical Analyses

Each N-of-1 trial data were summarized using the mean ± standard error (SE), and each of the data sets were summarized using the mean ± standard deviation (SD). For the crossover study, the Wilcoxon signed ranks test was calculated to examine the absolute differences in the DEHP removal rates by soap-and-water and water-only for the same individual. The Wilcoxon rank sum test used to compare the differences in the percent of DEHP removed. The same statistical analyses were also applied with data categorized by gender and/or experimental dates. All analyses were performed by one independent researcher who was blinded as to which group the samples belonged. P-values were 2-sided with a significance set at the <0.05 level.
