# Erratum: Removal of Diethylhexyl Phthalate from Hands by Handwashing: Evidence from Experimental N-of-1 and Crossover Designs

**DOI:** 10.1038/s41598-017-04860-w

**Published:** 2017-07-20

**Authors:** Pi-I. D. Lin, Chia-Fang Wu, Hwang-Shang Kou, Tzu-Ying Huang, Jentaie Shiea, Ming-Tsang Wu

**Affiliations:** 1 Kaohsiung Medical University, Department of Public Health, Kaohsiung, 807 Taiwan; 2 Kaohsiung Medical University, Research Center of Environmental Health, Kaohsiung, 807 Taiwan; 3 Harvard T.H. Chan School of Public Health, Department of Environmental Health, Boston, 02115 USA; 4 Kaohsiung Medical University, Department of Pharmacy, Kaohsiung, 807 Taiwan; 5 National Sun Yat-Sen University, Department of Chemistry, Kaohsiung, 807 Taiwan; 6 Kaohsiung Medical University Hospital, Department of Family Medicine, Kaohsiung, 807 Taiwan; 7 Kaohsiung Municipal Hsiao-Kang Hospital, Center of Environmental and Occupational Medicine, Kaohsiung, 807 Taiwan

*Scientific Reports* **6**:454; doi:10.1038/s41598-017-00581-2; Article published online 28 March 2017

In the original version of this Article, Pi-I. D. Lin, and not Ming-Tsang Wu, was listed as the corresponding author. Correspondence and request for materials should be addressed to Prof. Ming-Tsang Wu at e_encourage@yahoo.com. This has now been corrected in the HTML and PDF versions of the Article.

